# "The Hospital" Nursing Mirror

**Published:** 1901-04-06

**Authors:** 


"The Hospital" Nursing Mirror.

Being the Nursing Section of "The Hospital."

[Contributions for this Section of "The Hospital" should be addressed to the Editor, "The Hospital" Nursing Mirror, 28 & 29 Southampton Street Strand, London, W.C.]

### Notes on News from the Nursing World.

### ROYAL NATIONAL PENSION FUND FOR NURSES.

In order to correct an impression amongst certain policy-holders of the Royal National Pension Fund, that because at the time of entering they did not apply for sickness assurance they cannot subsequently do so, we are asked to state that a nurse already holding a pension policy, if eligible, can make a proposal for sickness assurance in connection with her existing policy.

### ONE OF GENERAL BADEN-POWELL'S NURSES.

Miss Flora K. Fitzmaurice has written to the directors of the City of Dublin Nursing Institution that she has received an appointment as nursing sister to General Baden-Powell's South African Constabulary. She therefore asks leave to resign her post as nurse in the Institution, "as her appointment will render it requisite for her to remain in South Africa for three years." The resignation has, of course, been accepted, and though the directors are sorry to lose the services of Miss Fitzmaurice, they are naturally gratified that she should have been singled out for distinction.

### THE ARMY SISTER AND THE BUSHMAN.

Among the exceptionally interesting patients of one of the sisters of the Army Nursing Service Reserve in South Africa was a man of Scotch and Irish parentage who had lived all his life in the pathless scrub of Queensland. The sister herself describes him as "the wildest man she has ever seen," and as such he was, she says, regarded by his mates and the soldiers, though a favourite with all—a great strong fellow of 6 feet, broad and muscular, with an absolutely fearless carriage and expression and a certain masterfulness about him which marked him out as a leader in mischief or heroism. Until he came to South Africa he had not worn socks, and only on rare occasions in the bush had put on moccasins of untanned leather. Generally, he had gone barefooted. He had never lived in a house, camping out first in one place, then in another, night after night. He would always sit cross-legged on the ground instead of on a chair by preference, and his bed—almost an unknown luxury to bushmen—was always the sister's despair. It could not be kept tidy, however often it was put straight. On first coming into hospital his language was very far from polite. Of course this could not be tolerated for an instant, and the sister spoke to the point quietly when alone with "Bushman." Not one bad word was spoken since. He pleaded that from the time he was three years old he had learnt to lie and to swear, so that the effort to abstain was a real trial. The only person who was able to subdue his temper was the sister, and her displeasure, incurred once or twice, had a most tranquillising effect, which was not entirely due to severe words. But he found it difficult to conform to rules and regulations. One morning coming on duty the sister was greeted with the information that three of her patients, among them "Bushman," had been seen in the garden, at 3 A.M., up the trees stealing fruit, and on being surprised had got into the ward again by the window. Expostulating with him on such shocking behaviour, and drawing a gruesome picture of the possible results of eating the pears, the sister elicited the candid statement "Oh, the pears wasn't worth eating, it was just the fun of stealing them."

### THE QUESTION OF PRESENTS TO NURSES.

A correspondent, who, as a nurse, protests against the practice of giving presents to nurses, will probably provoke remonstrances. It will be said, Why should not a well-to-do patient, or the friends of one, make a present to a nurse who has been of great service? Undoubtedly such presents have been made in the most delicate manner, and have been received in the spirit they were given. But the idea that a patient is expected to make a present to a nurse, in addition to her fee, should, of course, be strongly discouraged. There is really no reason why a nurse should be treated differently in this respect from a doctor, who never anticipates more than the payment of his fees. But there have been known instances of very grateful and generous patients insisting upon making an addition to even a doctor's fee. The exception in either case only proves that the rule is right.

### THE NURSES OF BELFAST UNION INFIRMARY.

The new scheme for the employment and training of nurses at the Belfast Union Infirmary will come into operation on the 1st of next month. At a meeting of the Belfast Board of Guardians a letter was read from the Local Government Board which, "while not approving of the scheme, offered no objection." In the circumstances, the scheme, of course, provides that, while "nurses are under the direct control of the superintendent, they are also subject to the general supervision of the master and matron, who are the superior officers." We observe that after three years' service a certificate, signed by the chairman of the Board of Guardians, medical officers, and the infirmary superintendent, will be given showing the qualifications and character of the recipient provided the engagement has been in every way satisfactorily completed. But no certificate of efficiency will be given for less than three years' service, nor until a qualifying examination has been passed. The grades are classified as follows:—Probationers serving their first two years, and until they pass a qualifying examination; assistants who have not less than two years' service, and who have passed a qualifying examination; charge nurses of not less than three years' training, fully qualified and certificated by regular examinations, and in charge of wards. Probationers will be charged an entrance fee of £5 5s. There is no salary for the first year, but they receive board and lodgings, and after three months' service indoor uniform if their appointment be confirmed, and outdoor uniform at the end of the year if conduct has been satisfactory. The whole of the rules and regulations appear to have been drawn up by Dr. M'Donnell, and they have been unanimously adopted by the Guardians.

### NIGHT NURSES AND NORTHERN WORKHOUSE INFIRMARIES.

The Poor Law Inspector for the north-western district, Mr. Jenner-Fust, notices in a report which he has just issued that, while the increase in the number of nurses in the workhouse infirmaries has kept pace with the increase in the number of sick, there is still "great need in several instances for more night nurses, while a further lessening of the number of paupers employed under the name of attendants is much to be desired." The details supply evidence in support of the inspector's statement. Thus, the number of sick in the Lancashire workhouses, exclusive of imbeciles and epileptics attended in special wards, on January 1st, 1901, was 9,374, and the paid nurses in charge of them numbered 569 by day and 175 by night, giving an average of 13 patients to each nurse and 148 pauper assistants in the sick wards. Again, in the Unions of Bootley, Cockermouth, Penrith, Whitehaven, and Wigton, there were 151 sick paupers with an average of 21 to each paid nurse and 28 pauper assistants; Westmorland workhouses contained 53 patients, and only two paid nurses, with seven pauper assistants, whilst in the remainder of the district which comprises the Unions of Sedbergh and Todmorden, in the West Riding of Yorkshire, there were 45 patients with two paid nurses and eight pauper assistants.

### ENLARGEMENT OF A LIVERPOOL NURSING HOME.

Last week the new wing of the Kirkdale Home of the Liverpool Queen Victoria District Nursing Association was opened by Sir Thomas Hughes. Prior to the ceremony, Mr. Herbert Rathbone explained how the work is carried on in the nursing home. The city, he said, is divided into different districts. For each district there is a nurses' home, with a lady superintendent. There are also ladies associated with the various homes who, taking an interest in the work, contribute to the funds, visit among the patients, and supply the extra nourishment and clothing sometimes required by the patients. Connected with the Kirkdale Nurses' Home there are a matron and a staff of eight nurses. The ladies of the district who assist in the work form a committee to manage the affairs, but they, in their turn, are subject to the control of the council, which meet at headquarters. It is ten years since the Home was opened, and the wing, just added at a cost of £700, comprises a much-needed appliance, a dispensary room, as well as further bedroom and other accommodation. Sir Thomas Hughes, in the course of his speech, paid a handsome tribute to the efforts of the superintendent, Miss Mills, who is also responsible for the training of probationers.

### LECTURE TO THE NURSES OF CHELSEA INFIRMARY.

Mrs. Hayward, better known to the nursing world as Miss Rolleston (Sister Elizabeth, of St. Bartholomew's Hospital), gave a most interesting lecture the other evening to the nurses of Chelsea Infirmary on her recent experiences as an army nursing sister with the Imperial Yeomanry at Deelfontein. The lecturer wore the uniform of an Army Sister.

### BRISTOL DISTRICT NURSES.

Although the claims of the Bristol District Nurses Society are warmly advocated by many public men, and the Lord Mayor of the city moved the adoption of the report at the annual meeting, the accounts for 1900 show a balance of £136 of expenditure over income. Moreover, the committee have already been compelled, from want of funds, to withdraw a nurse from one district, "in spite of great efforts to obtain support for her." As one of the speakers observed, "it is deplorable that the large districts of Bristol cannot each support a nurse." But for the generosity of Mr. George A. Wills the position would have been worse, for an anonymous donor, who for several years had supplied the salary of the nurse for Ashton Gate district, died last year. The example of Mr. Wills in making up the amount, should have the effect of stimulating the liberality of other citizens at a juncture when help is so greatly needed.

### A DEFICIT AT CHESTER.

It is curious that, in spite of a deficit of upwards of £100 in the working of the Chester District Nursing Home, the expenditure exceeding the receipts by nearly a fourth, the chairman at the annual meeting should have characterised the report and accounts as "very satisfactory." That the work done by the nurses attached to the Home is excellent is clear from all the speeches made on the occasion, and from the fact that a staff of five attended 729 cases and paid nearly 25,000 visits. But so long as the Home requires at least £100 a year to pay its way, we cannot see how the position can be described as "very satisfactory." In our judgment, such a state of things is far from creditable to a city containing so many well-to-do persons and so many prosperous artisans as Chester.

### MORE NURSES WANTED AT BRIDGEND.

At a meeting of the Bridgend Board of Guardians the other day, the medical officer for the Maesteg district said that "during the past thirty years there had been in his district many cases of extreme suffering, and possibly deaths, for want of proper nursing." He instanced a case a few weeks ago: "A young man died from typhoid, and the wife, who had three young children to attend to, nursed her husband day and night until her strength failed her, and his death was accelerated by want of proper nursing." The only result of this statement on the part of the medical officer seems to have been that "the Board postponed consideration of the matter." It may be hoped, rather than expected, that there will not be any more deaths from want of nursing while the Guardians are keeping the question in abeyance.

### PROGRESS AT KETTERING.

The District Nursing Association in the thriving town of Kettering continues to extend the scope of its operations, and last year there was a substantial increase in the work done. The number of new cases was 304, and of visits paid 7,506. Twice during the year an inspector from Queen Victoria's Jubilee Institute, with which the Association is in affiliation, visited Kettering, and expressed herself highly pleased with the work of the staff and the arrangements at the Home. There was a slight increase in the income from subscriptions, but the extra expenditure on the maintenance account, incurred by the addition of a third nurse, was mainly defrayed by a sum realised by a dramatic entertainment and a dance in aid of the organisation. We agree with the committee that funds for such a purpose should not depend upon chance efforts, but should be raised in the ordinary and less precarious way.

### HEREFORD NURSING ASSOCIATION.

After an existence of fourteen years the members of the Hereford Nursing Association have incorporated some new rules into the general rules of the organisation. There is nothing extraordinary in their character, and they should admirably serve the purpose for which they are intended. A sub-committee, consisting of ladies, has been formed to superintend the work of the nurses. The number of visits paid last year by the latter, of whom there are two, was upwards of 7,000, and the number of cases 346. The financial position is excellent, the accounts for the twelve months showing a balance in hand of £58. Arrangements are being made for the occasional employment, when necessary, of a third nurse; but the committee, who seem to think that an untrained person would suffice, will do well to keep to trained nurses.

### DISTRICT NURSING AND PAYMENT.

In the report of the Gateshead Nursing Association, which is affiliated to Queen Victoria's Jubilee Institute, and which shows an excellent record for last year, reference is made to the result of an effort to establish a system for the attendance of the nurses on patients who would be willing to pay. "So far," it is stated, "this arrangement has not been taken advantage of in more than two cases;" but the committee are of opinion that when it is more fully understood by the public "this important addition to the work of the Association will be much appreciated." Cases have come under their notice "where patients would gladly have paid a fixed amount for the attendance of a trained nurse once or twice a day." The general superintendent of the Queen Victoria Jubilee Institute has paid a visit to the new home, which was opened in Gateshead at the commencement of last year, and pronounced it to be a delightful possession.

### WOOLWICH DISTRICT NURSES.

There was a great falling off last year in the support given to the Woolwich, Plumstead, and Charlton Nursing Association by the clubs and societies, due, it was said at the annual meeting, to the diversion of their contributions to the various war funds raised in the district. It has, therefore, been necessary to draw upon the Endowment Fund in order to meet the expenses of the current year to the extent of £49. Apart from this, the report is satisfactory, and shows that a most useful work has been done by the nurses, especially amongst the families of soldiers and sailors at the front. The number of cases nursed in the year was 704, which entailed an average of twenty visits each, the expenditure being less than £1 per head. At the close of the business proceedings, Miss Honnor Morten gave an interesting address on "Health Problems from a Nursing Point of View."

### A USEFUL INSTITUTION.

At the annual meeting of the Trained Nurses' Institution at Leicester the reports of the committee of the district and of the private nursing branches were adopted. The former showed that during the year an increasing confidence in the district nurses was manifested by members of the medical profession, who had more freely recourse to their services in operations that could be safely performed in the homes of the poor. Although one of the speakers stated that the finances are not in a flourishing condition, there was a balance in hand of £63, and in the course of the year a legacy of £500 had been received under the will of the late Miss Dalton. This it has been very wisely decided to place to the credit of the reserve fund. The private nursing branch was sufficiently prosperous to enable the committee to increase the stipends of the staff, and to present a bonus of £5 to the lady superintendent and £2 to each of the nurses. A feature of the report of the private branch is that the lady superintendent could not on many occasions supply the public with nurses, although there are upwards of thirty on the staff. In January alone no less than 117 cases were necessarily refused; yet the number of cases nursed in 1900 was 273, as against 112 in 1899. We are glad to observe that a three years' hospital training is invariably insisted upon, and that the share of the legacy left by Miss Dalton has been invested with the view of applying the income to augment the nurses' sick fund.

### A DEFICIT AT GRIMSBY.

At the annual meeting of the Grimsby and District Nurses' Institution the chairman, in moving the adoption of the report, drew attention to the fact that there was a loss of about £120 on the year's working. He did not seem to think that there would be much difficulty in making up this amount when the public were made aware of the need. But why, if there is such a readiness to support the institution, was not a successful attempt made to present a satisfactory balance-sheet? It should always be the aim of a nursing organisation to avoid the declaration of a deficit. Another speaker intimated that the Board of Guardians do not contribute as largely as they ought, and if it is true that the poor are being attended by the nurses at a cost of one and five-eighths of a penny per visit, the association merits liberal support from the Board.

### THE PEPTONISED WARD-MAID.

There had been quite a stream of dyspeptics, gastric ulcers, and unhappy-stomached babies in our ward, and we seemed to be always in the "ward kitchen" preparing peptonised milk. Even the ward-maid caught the sound of the word, and used it when occasion required. "Ah!" nurse was saying, as she carefully warmed baby's "peptonised milk," "we shall never get another matron quite like *her*." "Never," agreed the other, mixing a "Fairchild's Powder" into its allotted "pint"; "and the best of her was you never heard her scold anyone; but she could *petrify* one with a *look*." Later on in the day nurse hurriedly entered the ward-kitchen. Our ward-maid was saying to another who had surreptitiously run across to "borrow," "You'd better not let matron see you here." "Why? What will she say?" "*Say!* she won't say nothing; she'll just stand in the doorway and *peptonise you with a look!*"

### SHORT ITEMS.

The new nursing home at Florence—the Villa Regina Natalia—has, we hear, already been of use to several English and American visitors who were taken ill in hotels. The nurses, of whom there are four, are hospital-trained Italians.—Miss Fitch has resigned her position on the workhouse infirmary staff at Skipton in order to enter a hospital for three years' training. — The Ipswich Guardians have increased their subscription to the Nurses' Home in the town from £10 10s. to £15 15s. per annum.—At a meeting of the Dublin Nurses' Club last week Dr. McDowell Cosgrave gave a lecture on "Animals in the Zoo and Out of it." Miss Huxley presided, and there was a large attendance of nurses, all the city hospitals being represented.—Miss K. A. Smith, whose appointment as matron to the Oxygen Hospital was announced last week, was trained at the Royal Infirmary, Arbroath, N.B. For the last three years she has been engaged in private nursing.—Nursing Sister Beaufoy is reported to be dangerously ill, at Germiston, of peritonitis, and Sister Dutton, who is at Springfontein, is also in a critical condition. A slight improvement is reported in the case of Sister Massey.

### Medical Lectures to Nurses.

By CARSTAIRS DOUGLAS, M.D., B.Sc., F.R.S. Edin., F.F.P.S. Glasgow, Professor of Medical Jurisprudence, Anderson's College Medical School, and Dispensary Physician, Samaritan Hospital, Glasgow.

### IV. PARALYSIS AND CONVULSIONS.

IN addition to coma, we frequently notice that the subjects of disease of, or injury to, the nervous system are affected with paralysis or convulsions. These conditions are so important and occur in the course of so many different illnesses that they are well worthy of some detailed notice.

*Paralysis.*—To define this in a wide sense we may say that paralysis is loss of the power of motion or of sensation (or both). When the function of a nerve is gone, and it can convey neither motor impulses down from the brain, nor sensory messages upwards, we say it is paralysed. Occasionally the term "paresis" is used to indicate a partial loss of power. Having defined our term, it will next be useful to consider briefly the physiology of the nervous system, as regards motion and sensation at least. The outer layer or cortex of the brain is composed of many millions of nerve-cells which, in certain parts, are gathered together into groups, to form what are known as "centres." There is, for example, a centre for the movements of each arm, of each leg, of the trunk, of the lips, and so on; these are motor centres. Nerve fibres arising from these centres pass down into the deeper parts of the brain, become collected together into a bundle, and at last reach the medulla, or beginning of the spinal cord, where they cross over to the opposite side to pass down the spinal cord. Eventually they emerge by means of the spinal nerves, and so are distributed to various muscles. And since the fibres cross over in the medulla, it comes about that the muscles on the right side of the body are governed by nerve-cells on the left side of the brain, and *vice versâ.* As regards sensation organs, sensations of touch, pain, heat, &c., pass upwards from the skin by nerve-fibres, which enter the spinal cord by means of the sensory roots; their subsequent course is upward through the cord and medulla, crossing over in the latter region (just as the motor nerves did) to reach the opposite side of the brain, and to end in clusters of nerve-cells forming sensory centres. We thus can understand that many different lesions may lead to paralysis. The mischief may lie in the brain, the spinal cord, or the nerves themselves. If the illness or injury be in the brain, the paralysis shows itself on the opposite side of the body to where the lesion is. Cerebral hæmorrhage, or ordinary apoplexy, is a good example of a brain affection causing paralysis. In such cases very often the whole of the opposite side of the body shows loss of power, and the name given to this condition is "hemiplegia." If, however, a special centre is picked out, say, the arm centre on the left side of the brain leading to paralysis of the right arm alone, the patient is said to have a "monoplegia." When disease attacks the spinal cord, leading, as it not unfrequently does, to paralysis of both lower limbs, the term "paraplegia" is employed, and where loss of power results from injury to an individual nerve, we sometimes call it paralysis of peripheral origin as contrasted with central. The chief affections of the brain that lead to paralysis are:—Hæmorrhage, embolism, abscess, tumour, meningitis, and pressure and damage due to fracture of the skull. In the spinal cord it is associated with meningitis and myelitis, locomotor ataxia, infantile paralysis, disseminated sclerosis, and disease of the vertebral column. In lead paralysis we have an example of loss of power due to disease of the nerves themselves. A nurse's chief duties in connection with a paralysed patient are the administration of food, attention to the bladder, bowels, and skin, the prevention of bed-sores, and the carrying out of the medical man's instructions regarding massage, electricity and exercises.

*Convulsions.*—By convulsions we mean sudden and involuntary contractions of the muscles of the body, often irregular in their rhythm, and of all degrees of violence. Sometimes one limb only is affected, as in Jacksonian epilepsy; in other cases the face may be the chief seat, as in certain cases of chorea and convulsive tic, while in not a few instances the whole body is thrown into violent contortions as in epilepsy, strychnine poisoning, puerperal eclampsia, and chorea occurring during pregnancy. In popular language when a person has a convulsive seizure, especially if there be also loss of consciousness, he is said to have a "fit," and very frequently this term is used by the laity in describing such attacks. It covers, however, so wide a range that we cannot be sure what has really occurred without going carefully into the case. The following are the leading morbid conditions in which convulsions are met with:—Cerebral hæmorrhage, cerebral embolism, and anæmia and congestion of the brain; tubercular and other forms of meningitis; tetanus or lockjaw; strychnine, alcohol, and other poisoning; epilepsy and chorea; certain general diseases, as chronic Bright and puerperal eclampsia, hysteria, and where sources of reflex irritation exist—*e.g.* threadworms. One of the most common varieties of "fits" is the hysterical seizure of young women. This may often closely simulate a convulsive seizure due to disease, especially an epileptic attack, and it is important to be able to discriminate between the two. In true epilepsy, males are affected as often as females; the onset is sudden; if there is a cry it is only at the commencement; the tongue is often bitten; the patient frequently passes water unconsciously; the fit lasts but a few minutes, and terminates spontaneously. In hysteria, on the other hand, women are usually the subjects; the onset is gradual; the patient may scream all the time; the hands may be bitten, but not the tongue; the patient never passes water; the attack may last ten minutes or longer, and may be brought to a sudden ending by a liberal sprinkling of water. In all genuine attacks of convulsions the nurse must first of all guard against the patient doing himself bodily injury; in addition, all tight garments and collars should be removed from the chest and neck, care should be taken that the nostrils and mouth are quite unobstructed, and the patient should have an abundant supply of fresh air.

### To Nurses.

**WE invite contributions from any of our readers, and shall be glad to pay for "Notes on News from the Nursing World," or for articles describing nursing experiences, or dealing with any nursing question from an original point of view. The minimum payment for contributions is 5s., but we welcome interesting contributions of a column, or a page, in length. It may be added that notices of entertainments, presentations, and deaths are not paid for, but, of course, we are always glad to receive them. All rejected manuscripts are returned in due course, and all payments for manuscripts used are made as early as possible after the beginning of each quarter.**

### Nursing at Friedrichshain Krankenhaus, Berlin.

By an English Nurse.

The hospital stands in the middle of a park, and has itself large and well-shrubbed grounds. It consists of pavilions, and, when the two new pavilions are ready, will be able to accommodate 900 patients; at present there are fourteen pavilions.

### The Wards.

Besides the different wards—medical and surgical—there is a diphtheria ward, and one for measles, &c. Leading out of each ward, which contains from thirty to forty beds, is a smaller room for convalescent patients (or, if very full, for extra beds), and that again opens on to a balcony, where some of the patients sit in fine weather; and sometimes beds are put out there in the daytime. There is also a small ward for one bed, a ward kitchen, and a room where the "ober-schwester" sleeps at night and sits in the day. In the corridor on one side is the steriliser, and on the other what looks like a huge drain-pipe, where the dirty linen is put down into the cellar beneath, being taken away every day by the men.

### The Operating Rooms.

The "operations-gebäude" or pavilion consists of three operating rooms, one being kept for septic cases only. The largest room is tiled, and has a large ventilator and a skylight in the ceiling. Operations are held from 10 to 2 every day but Thursdays, and the staff are most courteous in allowing any strange doctor or nurse to be present. I arrived at 10 one morning, having been the day previous to make enquiries and get the requisite permission, and I was asked to take off my bonnet; then, as I had provided myself with a cap and apron, I donned them, and was shown into the operating theatre. There the doctors and nurses were busy preparing themselves and the dressings. When they were ready they were covered with large long-sleeved aprons by a woman who did the work of a scrubber and junior pro. combined. She prepared the various solutions and handed them to a nurse, sterilised any instruments dropped on the floor, and, after the operation was over, washed the operating table with a disinfectant and also poured some over the floor under and round the table, and with a long rubber broom (like those used in the streets for mud) swept away the dirty sponges, &c. A nurse I had seen the previous day introduced me to the ober-schwester, who spoke to the head doctor about me and then told me to go and speak to him myself. I felt rather nervous at first about talking German to him, but very soon he made me feel quite at home, and we chatted together quite freely. Then he introduced me to another doctor who could talk English, and presently in came another stranger, an American doctor visiting Berlin in order to get an insight into the working and method of German hospitals, &c. There was also an Egyptian doctor from Cairo.

### Treatment of the Patient.

What struck me most in the operating room was that during the washing and sterilisation of hands, the preparation of instruments, and the gossip, the patient, a middle aged woman, was already on the table, and practically nude—that is, she had on only a sterilised cloth over the upper part of her chest, and another over her knees (the operation was for umbilical hernia), the arms and lower legs being wrapped in cotton wool and bandaged. It made one shiver to think of the poor woman's feelings, for besides the exposure for so long, and the dread of what was to come, the theatre was very cold, and there was an open window through which a wind blew down every time the door was left ajar which was rather often.

### The Staff and Diet.

Before all the doctors arrived I managed to have a little talk with one or two of the sisters, and asked them about their work. The staff consists of the matron, three directors (doctors), resident and non-resident doctors, and seventy nurses. Each ward has an ober-schwester or sister, two staff nurses, and a ward maid (if a male ward it has also a male nurse to attend to the men) by day, and one staff nurse by night. The sister who took me round the various buildings was just off a month's night duty. The night sisters go on duty at 8 P.M. until 9 A.M. At 6 A.M. they prepare the breakfasts for the patients and have their own, viz. coffee and rolls. The morning's work is then the same as that in an ordinary English hospital. At 9 A.M. the patients receive bouillon and milk, at 12.30 lunch or dinner, and at 6.30 supper, when they have bouillon, &c. We went into the kitchen, where the food was being cooked in huge round copper cauldrons, the contents of one being energetically stirred with a long pole. Observing my interest in the proceedings, the head cook informed me that it was rice and milk, which, with sausage, &c., was for the dinner of those patients on ordinary diet. As we went round one of the female wards we saw a nurse cutting big slices of brown bread, then buttering it and putting on what I thought was jam, but which was the raw meat portion ordered for some of the patients. The nurses' food is prepared and eaten in the "Victoria Haus," which stands at the other end of the garden across the street. As I had been told of one hospital in Berlin where the nurses only got meat twice a week and live principally on rice and stewed prunes, I made enquiries about the food here, and find that they are well fed, with two hot meals in the day, midday and evening. The day nurses work from 6 A.M. till 8 P.M., with two hours off duty daily.

### Four Years' Training.

The course of training is four years. Each nurse pays £16 to the Victoria Haus on coming to the hospital, which is returned at the end of four years. If a nurse leaves before that time she forfeits it. For the first six months nurses are "schülerinnen"—that is, having lessons from the doctors twice a week in the various subjects and also in the wards doing junior pros'. work; they do nothing for the patients. After six months they begin ward work proper—making beds, &c. They are then paid 9s. per month for the next six months. At the end of the year they are paid £1 a month, which continues until the termination of the four years, when they receive 4s. more a month until £25 is reached. This is the limit of payment, and they have two hours daily off duty—12 to 2 or 2 to 4, and from 2 to 11 P.M. once a week and 4 weeks a year. Those who have received their training are given a certificate and wear a silver chain round their necks with a crown and a large monogram, V.H. (Victoria Haus), suspended from it. The laundry is worked by machinery, with the latest up-to-date methods for economising labour. The head laundress told me that she has 19 under her, and that every day at 2 P.M. the dirty linen averaged 16 cwt. Besides the ward linen there are the nurses' things, the doctors' and the "dieners'" overalls.

### Lectures to Head Sisters.

BY MISS E. MARGARET FOX, MATRON OF TOTTENHAM HOSPITAL.

### LECTURE I.—A HEAD SISTER'S DUTIES TOWARDS HER PROBATIONERS.

(*Continued from page* 335.)

By the end of her first week your new probationer will be quite accustomed to the routine of the ward, and should know how to make a bed, how to sweep and dust properly, how to fill hot water bottles, wash convalescent cases, dress and undress a patient, know where all the things used in the ward are kept, and how to serve round all but the special diets. She should not be allowed to wash bad cases, help with dressings, nor on any account be left alone on duty. Routine duties having become familiar to her, she will most likely be very glad to be told other things, and if you take the opportunity in the evening, or any other time when the ward is quiet to give your new comers a little instruction we shall hear less about sisters having "no time" to teach.

Do not send her to fetch instruments, splints or dressings she does not even know the name of, while the doctors are in the ward. Sisters are rather apt to forget that in all probability a new probationer has never heard the word "iodoform" for instance, until she came here, so that it conveys no more meaning to her mind than did that "blessed word, Mesopotamia" to the old woman of apochryphal fame. Take it rather as a discredit to your powers of teaching, than that she is hopelessly stupid, should she make the same mistake more than once. Of course you must often make the best use you can out of unpromising material. You cannot alter the brain power you have to deal with, but you can show them how to use what they have to the best advantage. It is not possible for you to inculcate tact, good temper, sympathy, or common sense; but by example and precept you can do much to make a noisy person quiet, an untidy careless, person orderly and careful, by your steady insistence on the punctual, orderly performance of the routine duties of the ward. Let a new comer work at first largely under your own immediate supervision, When she has learned how to take a temperature give her a chart or two to keep, but look after it carefully yourself, and check her record of pulse and respiration with your own watch. Do not put her, the first thing, to work by herself in bathroom or ward-kitchen, cleaning utensils or washing up in the first way that occurs to her. Make a bed with her, show her how to wash a patient, and when she gives the water to a convalescent to wash himself, tell her about removing his shirt and hanging it up to air meanwhile. It will give you far less trouble in the end if you take pains to teach her how to do things right in the first instance, than if you are too lazy to do so. It is always easier to form a new habit altogether than to get out of a bad way of doing anything.

By the end of three months a probationer, without any previous training, should she remain as long as that in the same ward under a good teaching sister, ought to be very useful. In a men's surgical and accident ward, for instance, she should have learned at least these things. She should know how to keep a daily, four-hourly, and urine chart neatly and correctly, how to give bed-pans, sweep and dust, how to clean the various articles used in the ward, including test-glasses, mackintoshes and splints; how to wash a patient in bed, and prepare a bath for those able to take one; how to pad splints, and the names of those in everyday use; how to apply a fomentation; what to get ready for an ordinary dressing; how to pad crutches and roll plaster bandages. She should be able to prepare a bed quickly for an accident, and to get it ready for a patient who has gone to the theatre—also she should know what has to be done for a patient before he is operated on; how to feed helpless cases; how to remove clothes from a fractured limb, and what to get ready when a fracture or an extension is going to be put up.

If it is in a men's medical ward where she has made her start, she should know what to get ready for an "exploration," an "aspiration," a "tapping;" how to measure and test urine, and how to read the various simpler signs on the prescription papers; how to get the hypodermic needle ready for use; the best way of cleaning spittoons, and some of the most obvious facts to be learned from the sputum.

If she begins in the women's ward, she should at the end of three months have learned how to give a douche, pass the catheter, and how to bath an adult.

If in the children's ward, she should know how to wash an infant properly, how to "change" it, so as to prevent soreness and chafing. She should understand how to prepare and give babies food, and how to clean the feeding bottles.

In addition to these special bits of practical knowledge, only to be acquired in the special wards, a probationer should know at the end of three months, in any ward, how to prevent bedsores and to feed and wash helpless patients; how to prepare and give purgative and nutrient enemata; how to clean the heads of dirty patients, and what to do for a body after death. She should also have learned how to make barley water and peptonised milk, and should know what is expected of her as regards hospital manners and etiquette.

There are of course other things she should know, but which scarcely come under your province to teach her, such as signs of fracture, symptoms of various diseases, and other items which come under the head of observation of patients; but these, she will be taught definitely in the lectures she attends, while you are mainly responsible for teaching her the doing of the practical work.

Do not fall into the error of making your new probationers entirely responsible for anything. The strangeness of their surroundings makes it hard to remember things, and if the duties are forgotten, or badly done, it will be you and not they, who will be held responsible. Always require that they refer to you in everything and do nothing without leave.

I have known a new-comer loosen a splint after a fracture had been put up, under the impression she was doing the patient a kindness, because he complained of its being too tight! Lose no time in impressing on them that they must disturb no dressing, splint, or appliance put in position by the doctor on any account, no matter what the patient may say. Of course, that does not mean that the patient's complaint of pain is to be disregarded, but it must be reported, and the doctor, if necessary, will remedy it himself.

Besides your teaching responsibilities towards your probationer, you must feel responsible, in a way, for their health.

Just at first, they often do not know how to economise their strength, and so waste it in useless directions; besides which, the unusual strain of being on their feet so many hours a day is sure to tell upon them. Do not therefore send them on unnecessary distant errands, nor choose particularly laborious tasks for them. If the work is sometimes finished ten minutes before your probationer is due to go off duty, let her have the extra ten minutes if you think she looks tired, and give her a little sisterly advice about resting when off duty, about going out into the fresh air, and about taking food at the right times. Should she complain of not feeling well, it is always safe to take her temperature, and if raised, to report it. A "bad finger" should be attended to promptly, and she must never be allowed to be on duty with a sore throat or a rash of any kind. Never attempt to treat her yourself, nor give stimulants or medicine of any kind whatever without orders, unless in a case of extreme and sudden urgency.

A disregard of this positive rule—which, even if not in the standing orders of your hospital, you will do well to make for yourselves—may lead to serious results.

### Visit to a Cingalese Insane Asylum.

BY A NURSE GLOBE-TROTTER.

SEVEN O'CLOCK in the morning according to English ideas of time is a most unsuitable hour at which to visit a public institution. In Ceylon a before-breakfast round of a hospital or asylum is quite "the thing." Later on the stifling heat makes an up-and-down stair visitation a penance and weariness to the flesh. Unlike most tropical institutions, which are commonly built on one floor, bungalow fashion, the huge Insane Asylum at Colombo possesses many stairs and an upper storey.

Accordingly, at 7 A.M. punctually, after a charming drive through the lovely Cinnamon Gardens, which are the joy of generations of globe-trotters, one of the highest officials of Ceylon and I explored the wilderness of tropical trees, flowers and gorgeous blossoms, in which the vast Insane Asylum of Colombo, containing some 1,200 patients, stands. The patients, like ourselves, had taken an early cup of tea and bread and butter, the more substantial breakfast which we subsequently saw in process of being cooked is served at about 10 A.M. It was the Europeans who taught the Cingalese to drink tea, a habit which has now become inveterate and exaggerated in native family life.

The asylum household had been up and busy for about a couple of hours before our arrival. Groups of semi-safe patients were scattered under the shade of the trees, for even at this early hour the sun was sufficiently strong to make a shelter from its rays both grateful and comforting.

They were squatting, native fashion on the grass, chattering, crying and laughing in turns. Most of the men were smoking, some were dressed in the Cingalese "half petticoat," wearing turbans made from several yards of linen wound about their heads, others were garbed coolie fashion in a single wide loin cloth.

### A DRAWBACK TO GOOD NURSING.

The native ideal of bliss is to squat eternally—and talk eternally. Before visiting the East, I had accepted the "grave quiet Oriental" of literature as a gospel truth, but as a matter of fact, neither the man nor the woman of the East ever stops talking. They stay awake most of the night, because the day has not been long enough to give them time to say all they want to. And if by some chance sleep overcomes a native he still goes on talking. This incessant chatter is a terrible drawback to good nursing, both in Ceylon and India. For the nurse finds it a most difficult matter to obtain absolute quiet for her patient, either in the daytime or the still small hours of night. The punkah puller's wife and family cannot be prevented from camping out with him day and night, so as to form a cheery conversational party. The incessant prayer of a patient in the East is for one hour's real rest. For if the humans can be silenced by superhuman effort, threats and fines, the flying foxes, monkeys, jackals and crows all have their noisy innings till a sick man's nerves grow raw with noise.

### AMUSEMENTS.

The amusements of a Cingalese Asylum are few and far between. Natives play very few games, though they are incessant dice throwers and gamblers. Amusements such as are common in the home asylums would not be a success if they were introduced in the East. None of the inmates would know what to make of a piano. The only form of music appreciated by the Cingalese is that resulting from the tom-tom and a gruesome imitation of a bagpipes. All other harmonies would be regarded as weary, stale, and unprofitable. Since the beating of tom-toms could hardly be permitted—for the sake of the sanity of doctors and nurses—the routine seemed somewhat dull from the European standpoint. But the native does not find it so.

### THE NURSING STAFF.

The matron and all the head nurses are Burghers, which means that they are of Dutch extraction with a more or less co-mingling of native blood. The Burgher of Ceylon corresponds to the Eurasian of India. There is a large staff of Cingalese attendants in addition, who do duty of a non-nursing kind. The patients help to cook, do most of the domestic housework, keep the grounds in order, sew and assist the native and quite sane cooks. Some of the patients, recognising the important official who accompanied me, removed the cloth from their shoulders, this being the highest mark of respect to his superiors that a native can show.

### HOMICIDAL PATIENTS.

Many of the patients are homicidal, but the medical superintendent told us that epilepsy and epileptiform varieties of insanity are not nearly so common in Ceylon as at home. An old lady was pointed out to us whose greatest boast and pride in life rests in the fact that she cut her husband literally and truly into tiny shreds. She looked very crude and primitive as most of the Cingalese do, but there was nothing markedly ferocious about her appearance. Among the other patients she is a kind of heroine from the complete and unusual nature of her crime. The Cingalese temperament is rarely violent or cruel, though it is treacherous to a degree. It is a somewhat curious fact that the insane male natives we saw looked far more manly and decided than sane Cingalese men usually do. For it is a proverbial truth that the average Cingalese man looks "rather more like a woman than a real woman does." It was very touching to see an old insane native woman bestowing such tender care on her blind, insane, and grown-up daughter, whom she tends and watches and cares for as though she were an infant. Maternal devotion is characteristic of the native of Ceylon, and cruelty or even mild unkindness to children is very rare in the island.

### COOKING AND THE DIET.

The kitchen presented a novel scene. All the cooking—and capital cooking, too—is done picnic fashion, by means of a few smouldering logs and primitive saucepans. An old man patient—and we were relieved to hear that he was not classed with the homicides—was brandishing a big knife and shredding some fifty fresh half cocoanuts for use in the breakfast curry. After the nut is cut in fine shreds, it is rolled with the most crude kind of native rolling pin, before it is added to the curry-pot. Other patients were busily slicing the Bombay duck and somewhat "high" salted fish, without which and some twenty other odd vegetable and pickled compounds the native curry of the country would not be considered complete. Nice-looking rice "hoppers" were in process of preparation, the rice previously boiled being mixed with cocoanut oil into a consistent mass and fried, in small portions like pancakes or fritters. It takes a native to swallow, enjoy, and digest the strong rancid cocoa-nut oil which enters so largely into Cingalese cookery. Some of the patients were lepers, for leprosy is not yet compulsorily segregated in Ceylon. Many have become insane through immoderate drinking of the strong and deadly arrac spirit or toddy which the native distils so cleverly from cocoa-nut flowers. Indeed, he is very skilful at extracting strong drinks from almost any tree.

### GOVERNING BY KINDNESS.

The cubic space for each patient, judged by the British point of view, is enormous, but, of course, more air space is needed in the tropics. Very little restraint is used and a strong effort is made to govern by kindness and moral suasion rather than by a show of force. The patients appeared to be devoted to their doctors, some of whom are English while others are "burghers," who have mostly taken their medical degrees at Edinburgh. One old man, who was rather a bad case, hearing of our contemplated visit, determined to honour the occasion by gathering the most gaudy and resplendent tropical blossoms from the grounds. In the absence of flower vases he had filled two pairs of shoes, borrowed for that purpose from a European source, since the native wears only the hardened skin shoes wherewith nature endows him. Bright with tropical blossoms most tastefully arranged, four shoes stood ranged outside his cell door, and he proudly did the honours of the flower show. He had calculated on a money result—and he got it. It is impossible to visit and study the workings of this huge Insane Asylum of Colombo without being impressed with its admirable methods. Indeed, the asylum ranks high in the estimation of all who are familiar with it, and it is popularly allowed to be "a credit to Ceylon."

### Appointments.

Bradford Royal Infirmary.—Miss Caroline L. Jenkins has been appointed Home sister. She was trained at the Bradford Royal Infirmary, and at the end of her training was appointed sister, which post she held until January, 1900, when she became night superintendent.

Bristol General Hospital.—Miss Gummer has been appointed night superintendent. She was trained at Liverpool Royal Infirmary, and has since been sister at Bangor Hospital and superintendent nurse at Bristol Workhouse Infirmary. She holds the L.O.S. certificate.

Eastville Workhouse Hospital.—Miss Sarah Ellen Garrett has been appointed charge nurse. She was trained at Newport Union Infirmary, and has since been assistant and charge nurse in the same institution.

King's Norton Union Infirmary.—Miss Elsie Price and Miss Mary Sharp have been appointed sisters. Miss Price was trained at Brownlow Hill Infirmary, and has been charge nurse at Worcester Infirmary. Miss Sharp was trained at Chorlton Union Infirmary.

Mengo Mission Hospital, Uganda.—Miss Beatrice Dallison has been appointed to take charge of the nursing arrangements under the Church Missionary Society. She was trained at the Stanley Hospital and Mill Road Infirmary, Liverpool.

Park Hill Hospital, Liverpool.—Miss Catherine Marie Duffy has been appointed charge nurse. She was trained at the Royal Albert Edward Infirmary, Wigan, and has since been staff nurse at Park Hill Hospital, Liverpool.

### Presentations.

Burnley Union Infirmary.—Before leaving for Portsmouth, Miss Hall, late superintendent of nurses at Burnley Infirmary, was presented with a silver cake-basket by the staff as a token of the high esteem in which she is held. At the same time the senior sister, Miss Arnott, who is also leaving, received a somewhat similar present, both of them bearing the inscription: "Presented by the Burnley Infirmary Staff, March, 1901." A silver cake-knife was given to Sister Arnott by the patients in her ward.

### Where to Go.

Reading in aid of the funds of the Hammersmith and Fulham District Nursing Association Tuesday evening, April 30th, at Carnforth Lodge, Broadway, Hammersmith, by Mr. James Rhoades. Subject: "Betsy Lee," by the Rev. T. E. Brown.

### An Appeal for Trained Nurses in the Mission Field.

By a Correspondent.

There has recently been held in Preston a missionary exhibition at which I was in charge of the operating theatre shown in the Church Missionary Society's Medical Missionary Court. Besides myself, only one other trained nurse volunteered for duty. As there are a number of trained nurses in Preston, I infer from this that nurses as a body know very little of mission work, and do not take a general interest in it.

We had a model of a modern operating theatre, with instruments, operating table, instrument tables, sterilisers, &c., all lent to the Church Missionary Society Medical Missions Auxiliary by a London firm. The object of course was to give people an idea of what we want to send out to our mission hospitals, and also to show how the money given is spent.

There can be no doubt in the minds of any thinking man or woman that medical mission work is one of the most likely ways to reach the hearts of the heathen. In Turkey and Palestine an ordinary clergyman would not be allowed to get up in public and preach the Gospel—at least, he would run great risk, possibly even of his life. But in all lands, heathen and Moslem alike soon learn that the European physician can and will relieve his pain and discomfort, and when this has been done he will then listen to what the missionary doctor has to say about God.

There is no such thing as love in a heathen religion. The basis of our religion is love; the only way to teach love is by practice, and the first step towards love is gratitude.

The medical missionary is very much hampered in his work if he has no trained nurse to assist him. There is an enormous work for trained nurses in the mission field, and glorious opportunities of working for the Master. Women and children are dying by thousands from pure ignorance and neglect. For Indian women much has been done and is being done by the lady doctors, but the missions in Central Africa, Palestine, and China want nurses to help the doctors, not only in their medical and surgical work, but in their religious work, too. Nurses can do so much, not only by direct teaching, but by their lives—the patience, gentleness, good-temper, ever-ready obedience to doctors and superiors, and willingness to help and comfort the patients themselves, are all so many lessons of love. Midwives, too, are very badly needed. The awful ignorance about new-born babies and the resulting treatment is appalling. In Mengo, for instance, as soon as a baby is born it is laid on a plantain leaf, and cold water poured over it. The result may be left to imagination. Or fancy a poor little eighteen-months-old baby suffering from ordinary dentition fever being scarified all over its body by a native doctor in order to relieve the fever. Our doctor was called in after this had been done, and naturally found the child dying from sheer anæmia. Some of the diseases most common abroad are malaria, small-pox, dysentery, meningitis, nephritis, and ascites—all of which naturally require skilled nursing. There are a good many trained nurses in England, many of whom are good religious women with no home ties; will not some answer the cry from the mission field and offer themselves for the work? Any fully-trained nurse applying to the Church Missionary Society, Salisbury Square, will be able to get any and all information she needs, and will be heartily welcomed.

### Examination Questions for Nurses.

### THE TREATMENT OF BURNS.

### Result of March Competition.

The question was as follows:—"What are the chief points to be attended to, and what dressings should you prefer to apply, in a long case of extensive burn?"

### The First Prize.

"Nurse Mollie" wins the first prize because she recognises the dangers to internal organs, as well as the tendency to contraction. Almost all the competitors advocate the use of boracic lint and ointment, and undoubtedly they are most useful agents, but are they sufficiently aware that there are many skins on which boracic acid has a deleterious effect, producing a slightly inflammatory kind of eczema? It needs careful watching.

### The Second Prize.

"Paddie" is the second successful candidate. She would have come out first if she had mentioned the serious internal dangers to be apprehended, for she has noted intelligently the fact known to all who have much to do with burns, that it is most beneficial to vary the treatment continually. There comes a time when the most successful dressing seems to have lost its virtue and the sores make no progress, even if we are spared the mortification of seeing a retrograde condition set in. This is the time for a change of application.

Several nurses speak of red lotion and copper lotion as useful remedies, and they are both excellent aids at certain stages in the cases.

### Honourable Mention.

"Nurse Nellie," "Nurse Henry," and "Nurse Mary" have won honourable mention. May I remind you all once more that such names as Nellie and Mary are too common to be well chosen?

### First Prize.

The chief dangers to be apprehended and for which a watch should be kept in long cases of extensive burn are:—

(1) That the patient may succumb to inflammation of the internal organs even after the sloughs have separated whilst suppuration is going on.

(2) He may be worn out by exhaustion from the continued suppuration.

(3) He is exposed to the dangers of secondary hæmorrhage from the separation of the sloughs.

(4) Blood poisoning may set in from absorption of the discharges.

(5) There may be diarrhœa, whereby he will become exhausted.

(6) The kidneys occasionally become affected, so that a watch should be kept on the urine.

The patient's strength must be well maintained by a generous and nourishing diet, and, as a rule, it is advisable that he have some kind of stimulant.

Very good dressings are zinc or lead ointment spread on lint and applied in the usual way—*i.e.*, in strips—and lotions of lead, morphia and glycerine, or of sulphate of zinc. If there is much discharge and foul odour, iodoform is very useful, as it is found to soothe the pain and favour healing. When the sloughs are long in coming away, boracic fomentations may often be successfully applied. High granulations should be treated with nitrate of silver. All pains must be taken to avoid contraction, which leads to such hideous deformity, and when it cannot entirely be overcome, in the case of a limb, it should be kept in such a position as will be most useful to the patient afterwards. Nurse Mollie.

### Second Prize.

In nursing a long case of extensive burn, one must be careful, in dressing the wounds, to expose as little as possible at a time, in order to avoid shock by exposure to the air; and not to change the dressing more often than necessary, as the removal of the dressing causes intense pain. When the wounds are healing, one must prevent, as far as possible, the contraction of the skin which generally follows.

The patient's strength must be well maintained by nourishment and stimulant; and one must, of course, be watchful to prevent bedsores.

If the burn is one in which the epidermis is blistered only, at the first dressing I should snip the vesicles, irrigate with boracic lotion, and dress with some soothing antiseptic—boracic ointment, eucalyptus oil, &c.

In the case of burns in which the skin is destroyed, I should irrigate with boracic lotion, and apply iodoform emulsion freely on gauze, unless there were much suppuration, in which case I should apply boracic fomentations until the wound were cleaner, and then follow by iodoform.

Owing to absorption, it may not be possible to continue the iodoform for a lengthy time. I should then substitute carron oil, eucalyptus oil, or boracic ointment.

Sometimes, when one remedy has been applied for some time with little improvement in the condition of the wound, it may be well to vary the treatment; one may, perhaps, in that way discover what is the particular remedy which is suited to the patient's constitution or idiosyncrasy.

Nurse Paddie.

### Improvement in the Answers.

On the whole the papers are better this month. I am glad to note more intelligence in the manner in which the answers follow the question, instead of going off on branch lines, divergent from the matter in hand; also to see less tendency to the giving of unasked-for information. This latter fault is caused not so much by conceit as by an inability to arrange the ideas in a succinct and clear manner.

### Chief Faults in the Papers.

First, as usual, there is a failure to grasp the whole subject; even the two prize winners are examples of this. "Nurse Mollie" tells us of all the serious internal dangers and gives a good range of dressings, but fails to mention the benefit to be hoped for from a frequent change in their composition. "Paddie" is keenly alive to this, but fails to speak of evils not visible to the naked eye. Some papers are well thought out, but very carelessly written. "Busy Bee" says, "the edges cleansed by dabs!" One can only suppose she means "swabs!" "A Late District Nurse" advises croton oil as a dressing! She means, of course, carron oil; but such carelessness is criminal, and may cost a life should such an order be given to an inexperienced nurse. "Nurse F." says she should "advise quiet and rest in bed." A person suffering from extensive burns is not likely to be walking about or even sitting up.

### The Question for April.

If called to an operation, such as abdominal section, at short notice in a small house, what preparations should you make in the room and with regard to the patient? You need not consider dressings or lotions; they would be brought by the surgeon. The Examiner.

### Rules.

The competition is open to all. Answers must not exceed 500 words, and be written on one side of the paper only. The pseudonym, as well as the proper name and address, must be written on the *same* paper, and *not on a separate sheet*. Papers may be sent in for fifteen days only from the day of the publication of the question. All illustrations strictly prohibited. Failure to comply with these rules will disqualify the candidate for competition. Prizes will be awarded for the two best answers. Papers to be sent to "The Editor," with "Examination" written on the left-hand corner of the envelope.

N.B.—The decision of the examiners is final, and no correspondence on the subject can be entertained.

In addition to two prizes honourable mention cards will be awarded to those who have sent in exceptionally good papers.

### Echoes from the Outside World.

### AN OPEN LETTER TO A HOSPITAL NURSE.

It seems to be the intention to erect the Queen Victoria Memorial—the structure which is to be set up in front of Buckingham Palace—as soon as possible. Five of the leading architects of the United Kingdom have been asked to provide designs for a fitting shrine in which to place Mr. Brock's statue of her late Majesty, which has been already accepted by the committee, after approval by the King. Three of the competitors are English—Mr. T. G. Jackson, R.A., Mr. Aston Webb, A.R.A., and Mr. Ernest George, F.R.I.B.A. Then there is a Scotch representative—Dr. R. Anderson, of Edinburgh—and an Irishman—Sir Thomas Drew, of Dublin. By thus appealing to the three countries which form the Kingdom of Great Britain, the committee seem to have thought that they would please all parties, but complaints are being made that the choice should have thus been limited to a few, and suggesting that it is not too late to throw open the competition to all who lived under the Good Queen's rule. Decidedly to have allowed Greater Britain to compete would have been a very popular movement, and one which would have done much to insure the receipt of large subscriptions from the Colonies. Of course it is very easy to find fault, but the gain might have been worth the extra trouble involved.

Some of you may have read an account of the kindly concern exhibited lately by the Queen of the Belgians when an accident occurred in the neighbourhood of Laeken. Her coachman had the misfortune to knock down an old man. The Queen at once left her carriage to see if the poor man was hurt, and finding that he had evidently been badly injured, she caused the sufferer to be placed in her carriage and driven at once to the hospital, telling him that she would see that his wife and children were informed of the occurrence and were taken care of. Since then she has instructed her secretary to forward to the family 40,000 francs. This incident has produced a letter from a Belgian showing that, though an account of the kindness of the Queen on this particular occasion has crept into print, it is by no means a solitary example. The serious illness from which she is only just now recovering, and which was so severe that her life was for a time despaired of, was the result of the consideration and unselfishness she invariably manifests to those about her. One cold night she found herself unable to sleep, and thought that if she started knitting, it might possibly act as a soporific. The knitting was in an adjoining apartment, and as she was loth to waken her lady-in-waiting for what she considered such a trifle, she got up and slipped out to get the knitting herself. The sudden change to a cold apartment produced a dangerous chill, from which at one time it seemed probable her Majesty would not recover. A pleasing phrase in the letter is the concluding one. After the writer has pointed out that people in high places can show as much consideration as any in a less exalted station, we read, "This was especially the case with your Queen Victoria." One has become so accustomed to the inhabitants of Belgium, since the Boer dispute, saying hard words of anything English, that it is delightful to read such a true statement from a private foreign source.

An appeal for books and games for the use of the newly-raised South African Constabulary has been made by Lady Sarah Wilson. A letter which she quotes from General Baden-Powell will probably have excellent results. B.-P. says: "What the men specially want are books. All these sick and wounded ones are wonderfully patient, but their hunger is for something to read." He then goes on to tell how they managed to buy a truck-load of books, got a permit for the truck, and then, when it came within 25 miles of its destination, the Boers came down, derailed the train, and burnt the little library. And so he appeals for more volumes, with a few boxes of chess, draughts, halma, &c., for the use of the six hospitals now in working order and the dozen small ones which, with 97 hospital tents in various parts of the country, are yet to be erected. The recruiting officers—Major Hoare and Captain MacLaren, at the Chapel Place, Delahay Street—will undertake to get any books shipped out to the Cape, and Lady Sarah Wilson will spend any money advantageously which may be sent her to purchase library contributions.

The new Canon of St. Paul's, Mr. Cosmo Gordon Lang attracted the attention of Queen Victoria soon after his appointment to the important living of Portsea, and speedily became one of her favourite chaplains. Mr. Lang is not married, and his more than half-score of curates have resided with him at the vicarage. The story goes that the late Queen suggested that he might be happier with a wife, and that Mr. Lang, who has a sense of humour, replied that curates could be changed, but a wife would be a fixture. Nevertheless, as he is only six-and-thirty, the Canon may yet venture upon the experiment from which he appeared to shrink. He is a thoughtful and eloquent preacher, whose career in the capital will be watched with much interest.

It is seldom, even in this country of samples, that we have such cause to complain of the weather as during the month which has just ended. According to some of the learned ones of the earth a sun has burst somewhere or another, and the result has been, not only the red rain mentioned as having been seen in so many places, but the strange disturbance of the atmosphere which has brought us alternately snow, hail, thunder, frost, darkness, depression, downpours of rain and tornados of wind. Though I laughed, I could not help echoing the sentiments of a schoolboy friend who, hearing this explanation, expressed a hope that "someone would tie up the other suns jolly tight, so that such an accident might not happen over again." However, we may reasonably hope that April has better things in store for us, and that the evil spirits of last week, having done their worst, ending up in such an awful Boat-race morning, will allow the Fairy Good Weather to exercise upon us her sunshiny sway. I am grateful to say that I had no duty-call to go and see the Oxford and Cambridge contest, and I cannot imagine any sane person going for pleasure. That the race was such a close one makes it the more a matter of regret that so comparatively few were able to enjoy the keenness of the competition, but admirers of the Dark Blues maintain that unless their men had been out and out the best they could never have come in victors even by two feet with the worst of the river for nearly four miles of the course. Be that as it may, when the crews passed Barnes Bridge, where it used to be considered the contest was practically over, Cambridge led by a length and a half, but Oxford had kept a big reserve of strength which stood them in good stead. Both boats shipped a great deal of water, rain and river, and the crowds on the shore suffered considerably. Before the race on the river there were several races on land, mostly for runaway hats.

It seems probable that this year those who wish for hot-cross buns on Good Friday will have to walk some distance to procure them. The master-bakers, who like all the rest of the world in the present age are anxious to get as many holidays and do as little work as possible, have agitated to abolish the making of hot-cross buns on Good Friday, with the result that a large number of the trade in the metropolis have posted notices in their windows to the effect that hot-cross buns will not be made at their establishment. They will, I believe, have no objection to the making of buns on Thursday, which can be warmed by the consumers in their ovens on Friday, but the buns will no longer be procurable in a new condition on that day.

### English Nurses in a Train Attacked by Boers.

A CORRESPONDENT from Johannesburg writes:—"Few of the nurses in South Africa have had such an exciting time as Nurse Brading (civilian) and Nursing Sister Borlase, who not only went through the Ladysmith siege, but were in the mail train which was attacked by the Boers on the journey from Maritzburg to the Rand. They left the town at 10.20 P.M. and travelled through Natal during the night. At Newcastle, owing to a rumour of Boers being near the line, a train of Scottish Horse went on in front; but at Volkrust, as they had to feed and water their horses, the mail train proceeded without them. It was in charge of Captain Gilliatt, of the Imperial Yeomanry, and there were several doctors, as well as a few soldiers, returning from hospital to duty. After passing Greylingstad, it was remarked that the barbed wire fences were down and the telegraph wires broken; then suddenly there was a report like a shell; clouds of dust, showing the presence of dynamite on the line, surrounded the travellers, and the train came to a standstill. As the dust cleared the Boers could be seen coming down from the hills. The men on board at once opened fire, but there were only seven rifles in the train, and when about fifty yards off the enemy seemed to divide, and came on thicker and faster than before. The carriages were getting riddled, and one of the doctors called to the nurses to come out and try to take shelter in a small donga at the rear of the train. But there was no time, and as the luggage van was the next carriage to the one in which the nurses were travelling, fortunately their woman's instinct told them they would stand a better chance behind their boxes, which would probably receive the bullets! One gentleman got an explosive one in his thigh, eight others were hit, the stoker and another man were so badly wounded that they died the same night, Sister Brading had a bullet through her hat, and Sister Borlase had two through her dress. How they escaped without serious personal injury is a marvel, but in other ways they suffered severely. As soon as the ammunition had come to an end the Boers closed in, and all the occupants of the train had to put up their hands and acknowledge themselves prisoners. Then followed the scene of destruction. The Boers ripped open and took away in kit bags which they had brought for the purpose whatever plunder they could lay their hands on—money, valuables of any sort, dressing cases, and even big boxes. The Field Cornet promised that the nurses' property should not be touched, but whilst they were busy with the wounded everything was carried off. Happily, the firing was heard from Dene's Kop, and a shell was sent into the midst of the Boers, which resulted in them scampering away, or the nurses thought they would not even have been left what they had on. Their carriage had 38 bullets through it, of all sizes and shapes and kinds, and altogether they experienced a very bad half-hour."

### Everybody's Opinion.

[Correspondence on all subjects is invited, but we cannot in any way be responsible for the opinions expressed by our correspondents. No communication can be entertained if the name and address of the correspondent is not given, as a guarantee of good faith but not necessarily for publication, or unless one side of the paper only is written on.]

### THE QUEEN AND THE ROYAL NATIONAL PENSION FUND FOR NURSES.

"E. F." writes: Five members of the Royal National Pension Fund say in their letter that we (policy holders) bear the initials of their Majesties on our armlets. Do we really, or is the second initial simply a repetition for ornament's sake? Much discussion has been held on the subject by many of us.

### "TWO CONCEPTIONS OF THE NURSING VOCATION."

"AN ENGLISH NURSE" writes: When a matron of experience describes a nun as "an unsexed and unsympathetic nonentity," she shows herself lacking in sympathy with those who are daily performing works of mercy and charity. Nuns are ladies whose lives are a complete self-sacrifice. Go where you will—in the slums of London, in plague-stricken countries, or on the battlefield, there they will be found, nursing the sick and comforting with their sweet presence those who are in sorrow. Surely such lives are above criticism.

### A MIDWIFERY CASE.

"A. E. H." writes: Having had a peculiar midwifery case, and not being able to find an account of one like it in my books, the nearest being "missed labour" in Playfair, I thought I would like to write to you about it. I was called on March 11th; made P.V. Os was the size of half-crown. The patient had good pains, and went on very well for about three hours, when the pains went off altogether. The os then had nearly disappeared, and the membranes had ruptured. I visited her twice during the day, and again on the 12th, when I examined and found the os had completely closed up again. The patient was delivered of a fine female child on the 16th, after a normal labour of 8½ hours, and is now doing well. I hope that the case may get explained.

[Whenever anything unusual occurs a doctor should be called on it at once.—ED. THE HOSPITAL.]

### PRESENTS TO NURSES.

"NURSE" writes:—Your invaluable paper has done good service both to nurses and to the public. There is still a great grievance to many nurses which I feel sure you can do much to mitigate. It is the practice of giving presents to nurses. Why do patients and their friends think that they ought to give the nurse a present? They pay the fee she asks. It is a nurse's duty to do all in her power for a patient. Why should the patient think that he ought to give her more than her fee? He will say, why do nurses accept it? To the few presents are acceptable; to the many an insult. Nor is it always those people who can best afford it who give presents, but most often the patients who are hard pressed to make both ends meet. Education has done much to raise the social standard of nurses, but I think if the system of accepting presents were abolished it would do a great deal towards raising the moral standard. I feel very strongly on this point, for a few cases have come within my knowledge lately which made me feel anything but proud of being a member of the profession. Most nurses, I am sure, will welcome the day when accepting presents is a thing of the past.

### ALEXANDRA NURSES.

"LA BELLE MAURICE" writes from Mauritius: In all probability a nurse will be required for Mauritius during this year, and for the benefit of any nurse who may apply for the post I should like to tell you something about it. The nurse who comes here should be at least 30 years of age, qualified to do midwifery and general nursing, and willing to do either private, hospital, or district nursing as required. The work is a combination of all three, and there is very little of either. In the account of the Alexandra nurses it states. "No nurse is allowed to act as a midwife." Well, I must be the exception which proves the rule. As matron of the Female Hospital I always have to do so. Of course the medical officer will come to my assistance if I send for him, but I am not expected to do so except in abnormal cases. I have a nice little furnished house and garden with light and fuels, also an allowance for a servant, and rations. My life here has been rather dull and monotonous, but the climate is good and I have enjoyed excellent health. I am looking forward with great pleasure to a few weeks leave in England, but I do not regret coming, and shall hope to go to another foreign station. I should like to add that my salary is good and that until quite recently living was not expensive.

### PLYMOUTH UNION INFIRMARY.

Miss Holliday, Superintendent Nurse at Plymouth Infirmary, writes: Having read two notes lately in your columns regarding the Plymouth Union Infirmary, which might be misleading to your readers, may I be allowed to refer to them? In the first place I would say that there is no yard to be crossed by nurses except to the Lock and Isolation Hospital. The general wards are all within the one building. In one note you said that neither patients nor nurses could be comfortable; but that neither have anything to complain of. I have only recently entered on duty here, and must say that the Guardians have been most willing to do anything I have suggested, both for the comfort of the patients and for the nursing staff. The increase to the staff, three—two staff nurses and one male nurse—were granted to me when I asked for them the first week I took up my duties here. In fact, everyone connected with the Plymouth Union Infirmary has treated me with the utmost consideration and kindness. We are just about to begin building a Nurses' Home, and from what I have seen here, I am fully convinced that the Guardians are anxious to improve their infirmary in every possible way.

### THE ABUSE OF ANODYNES.

"A Saddened Nurse" writes: As a mental nurse of several years' asylum and general experience with patients suffering from weakened minds or mania from the alcohol or drug habits, may I most strongly protest against the giving into the patients' own hands such things as chlorodyne, laudanum for toothache, morphia for rheumatism, &c., or nepenthe for chilblains? At the present time the latter is being freely given to young girls at one of our southern towns. Very few persons at present know that the new drug nepenthe is a preparation of opium, and is sold as a "patent medicine." People take it on the recommendation of their medical man or friends without knowing its evil effects or properties. Some months since I had charge of a morphia patient, who ten years ago was advised to take the drug for nausea. Trying to break off the habit she added that of alcohol. At last, in despair, and after struggling in vain, she underwent the "Keeley" cure, and is now quite well. But everyone has not the means to undergo this great boon. Would it not, therefore, be better not to take to the drug in the first instance, unless administered by a doctor himself or a nurse? Then as to stimulants. How often one hears a "hot glass of whiskey" ordered for a cold, or "gin" for any kidney trouble. Not long ago a woman went into a chemist's and said "her back was bad," and never told him she had fallen off a ladder. He gave her an embrocation to rub in and told her to take a glass or two of hot gin and water. Ultimately the poor soul became so ill that she was taken to the hospital, and it was found that she had hurt her spine. For months she was an invalid. No one has cause to believe more in the value of drugs or stimulants than a nurse when given under medical advice and stopped at the right moment; but for nervous hysterical men or women who get a little depressed to be able to use them *ad lib.* is a ghastly idea and fatal in effect, as our madhouses, suicides, wrecked lives, and broken-hearted men and women can testify only too sadly. Unfortunately the nerve strain of our present life is so great that men and women working at "high pressure" and obliged to keep themselves up to concert pitch are only too ready to fly to fictitious strength, forgetting that after "ups" there must be "downs," and that if the alcohol or drug craze is once formed it is terrible for those whose will-power has failed to leave it off. As a nurse, therefore, who has seen many homes wrecked, wee children practically motherless and fatherless, debts, difficulties, estranged friends, and more than one suicide, I do beg anyone thinking of taking drugs or alcohol to pause, for once on that path there is no return, and the tortures are those of Hell itself. To no one do I write more than to nurses.

### The Nurses' Bookshelf.

Manuals of Employment for Educated Women.—No. III. Edited by Christabel Osborn. "Sick Nursing." (London: Walter Scott, Limited. Pp. 122. Price 1s.)

This is a carefully written and edited little book, and any woman desirous of taking up nursing in any of its branches would do well to consult it first. It is thorough and concise, and in a small compass takes in a wide range of nursing matters. Sentimental padding, a common fault in some works on nursing, is conspicuous by its absence, but there is much sound advice to nurses, notably on the care to be exercised in the choice of the line of work to be adopted, the differences between private and institutional work, the outlook for the future, the care of health, and the relative advantages of and points in connection with hospital, district, Poor Law work, and the services. Mention is made of perhaps the latest and least developed form of private nursing, namely, the daily nurse for patients who are unable or unwilling to have one in the house entirely, and yet are above the class for whom a district nurse caters. We would commend this branch of nursing to ladies possessing private means and leisure, in addition to a sound training. Another important point touched upon is the caution necessary in the choice of private nursing institutions, for as the authoress very justly remarks, "though many are well managed, there are, unfortunately, others of such a character that it can only do a nurse harm to be connected with." Miss Eva Lückes makes some good points in her pleasant and discursive introduction, notably one which is apt perhaps to get overlooked and under-estimated in an age of complicated technical instruction, but which to anyone acquainted with the practical, living side of nursing is daily more and more patent; in Miss Lückes' words, "no one wishes to under-rate the value of sound technical qualifications in these days. Indeed, anyone lacking these would not be deemed an eligible candidate for a trained nurse's post. But what I am anxious to impress upon every woman who means to become a nurse is, that in this perhaps more than in other professions, the most important factor in her success is her own personality." In conclusion, this little book is well arranged, well printed, and its information is under clear headings, which makes up for the absence of an index.

"The Zenana, or Woman's Work in India." The Monthly Magazine of the Zenana Bible and Medical Mission. Volume VII., 1899 and 1900. Office, 2 Adelphi Terrace, W.C. (Partridge & Co., 9 Paternoster Row, E.C. Price 2s. 6d.)

Twelve monthly parts make up this volume, which is neatly bound in blue-green cloth, and contains upwards of 200 pages. It forms an interesting record of a year's work done by women for women, and embraces accounts of what the devoted members of the Zenana Mission do in the way of teaching, doctoring, and christianising the native women of our Indian Empire. This is a work which must commend itself to everyone with a spark of benevolence, especially as those engaged in it carry on their labours in a quiet and unostentatious manner. Most of the monthly parts have a letter on medical work, and are dated from hospitals at Lucknow, Benares, and Patna; but the question of nursing is not gone into at all. A picture of a part of the Victoria Hospital at Benares shows a spacious, well-arranged ward, with a couple of women in native costume, who may, or may not, be nurses. White curtains, hanging from rods running across the ward seem rather a good feature, obviating the use of screens. This peep into the hospital makes us wish to hear a little about the method of working it.

### For Reading to the Sick.

EASTER DAY.

O DAY of days! shall hearts set free
No "minstrel rapture" find for thee?
Thou art the Sun of other days,
They shine by giving back thy rays:

Enthroned in thy sovereign sphere
Thou shedd'st thy light on all the year:
Sundays by thee more glorious break,
An Easter Day in every week:

And week-days, following in their train,
The fulness of thy blessing gain,
And in the ears of mourners say,
"Come see the place where Jesus lay!"

*Keble.*

On the Resurrection morning
Soul and body meet again;
No more sorrow, no more weeping,
No more pain!

Here awhile they must be parted,
And the flesh its Sabbath keep,
Waiting in a holy stillness,
Wrapt in sleep.

For a while the tiréd body
Lies with feet toward the morn;
Till the last and brightest Easter
Day be born.

Soul and body reunited
Thenceforth nothing shall divide,
Waking up in Christ's own likeness,
Satisfied. *S. Baring-Gould.*

Christ's death restores the lost life; and in restoring, effects an object higher than could otherwise have been. In Christ's sufferings there was more than the death of the body; a real spiritual life flows from His spiritual passion. He bore our iniquities, our death; from Him we thus derive new life.—*James Hinton.*

Glorious, indeed, will be the spring-time of the Resurrection, when all that seemed dry and withered will bud forth and blossom. The glory of Lebanon will be given it, the excellency of Carmel and Sharon; the fir-tree for the thorn, the myrtle-tree for the briar; and the mountains and hills shall break forth before us in singing. Who would miss being of that company? May the good Lord rouse the slumberers and raise them to a new life here, that they may inherit His eternal Kingdom hereafter!—*J. H. Newman.*

Let no man defraud you of your joy. We shall meet again even as we parted: yet not altogether; there shall be no more tokens of the fall, no more lines of sorrow, no more furrows of tears, no more distress, no more changes, no more fading, no more death; but all shall be fair, and radiant, and full of life, as in Him that said: "Behold . . . that it is I, Myself."—*H. E. Manning.*

A little while,
A little while, and we shall stand without
No more, to hear His voice; but enter in
With joy unspeakable, to see His face,

*Anon.*

### Notes and Queries.

**The Editor is always willing to answer in this column, without any fee, all reasonable questions, as soon as possible.**
**But the following rules must be carefully observed:—**
1. **Every communication must be accompanied by the name and address of the writer.**
2. **The question must always bear upon nursing, directly or indirectly.**

**If an answer is required by letter a fee of half-a-crown must be enclosed with the note containing the inquiry.**

*General Baden-Powell's Nurses.*

(1) I should be much obliged if you will kindly say to whom I should apply for service in General Baden-Powell's nursing corps at Pretoria. 2. Also for army nursing in South Africa?—*To L. A. H.*; *A. E. T.*; *Nurse C.*; *D. F.*; *Sister*; *C. H. H.*; *H. L. E.*; and *J. B. D.*

You must apply to the authorities in Pretoria. 2. See answer to F. M. lower down. "Sister" must advertise for travelling patient.

*Imbecile Girl.*

(2) Can you tell me where I could place an imbecile girl of 15 who lives in Lincolnshire? I have tried Earlswood, Royal Lancaster, and Eastern Counties, but to no purpose. Her mother is not in a position to pay, but I think there must be some place other than the County Asylum where she could be sent.—*E. A. T.*

We fear not, as the case appears to offer no hope of improvement.

*Baby's Bed.*

(3) The mattresses of the baby's cots in present use are frequently so hard that I should like to know what is the most comfortable and hygienic bed in order to advise young mothers?—*Nurse C.*

Make a mattress cover of swansdown calico the required size, and partly fill it with oat chaff to make an easy bed. The cover can be washed at frequent intervals and refilled with fresh chaff.

*Trained Male Nurse.*

(4) I have had six months' training in nursing medical, surgical, and infectious cases at the Royal Naval Hospital, Haslar; twelve months at Neville Hospital, Chatham; and an unbroken period of nursing experience from 1885 to May 1900. Yet the manager of one of the Nursing Associations implies that it is necessary that I should receive five years' further instruction in specified establishments before my qualifications would entitle me to recognition as a fully-trained male nurse. Can I not pass some examination which would testify to my being properly trained?—*John I. L.*

Yes, in consideration of your present training you would probably be allowed to present yourself for the examination of the Medico-Psychological Society. Apply the Registrar, 11 Chandos Street, W.

*Employment.*

(5) Can you help me in finding work, either writing or plain needlework, for a nurse incapacitated by varicose veins, &c.? She is most anxious to help to support herself.—*Constant Reader.*

The Secretary, Gentlewomen's Employment Club, 7c, Lower Belgrave Street, S.W., would be able to advise you, and advertisement might assist you.

*Address.*

(6) Will you please tell me the address of the Indian Hill Station Nursing Association?—*F. M. Keeble.*

Do you mean the Up-Country Nursing Association for Europeans in India? The address is Major-General Bcnus, The Cedars, Strawberry Hill.

*Consumption.*

(7) Can you tell me of a home where a poor deserving consumptive woman could be received, free, for treatment? Midland Counties preferred. *R.B.J.*

Either the Manchester Hospital for Consumption and Diseases of the Chest, Bowdon, Cheshire; or the Liverpool Hospital for Consumption and Diseases of the Chest, Mount Pleasant, might be suitable. Admission to the former is by payment in accordance to patients' means; to the latter, by a nominal charge with recommendation.

*Army Nursing Service Reserve.*

(8) Could you kindly give me the address of the Army Nursing Service Reserve?—*F. M.*; *Nurse G.*; and *I. L. G.*

18 Victoria Street, S.W.

*Teeth.*

(9) Can you kindly tell me if defective or artificial teeth debar a candidate from training as a nurse? I have found this the case with two well-known hospitals.—*M. A. G.*

Defective teeth are a great drawback, as toothache seriously interferes with a probationer's usefulness; well-fitting artificial teeth, on the other hand, provided they cover no malignant stumps, are not a disqualification.

*Maternity Fee.*

(10) I have been engaged since January 8th for a case that is still delayed. Will you kindly tell me if I can demand fees from that date? I have lost three good cases by it.—*A. S.*

You will of course be paid from the date of engagement, provided no arrangement to the contrary was made.

**Standard Books of Reference.**

"The Nursing Profession: How and Where to Train." 2s. net; post free 2s. 4d.
"The Nurses' Dictionary of Medical Terms." 2s.
"Burdett's Series of Nursing Text-Books." 1s. each.
"A Handbook for Nurses." (Illustrated.) 5s.
"Nursing: Its Theory and Practice." New Edition. 3s. 6d.
"Helps in Sickness and to Health." Fifteenth Thousand. 5s.
"The Physiological Feeding of Infants." 1s.
"The Physiological Nursery Chart." 1s.; post free. 1s. 3d.
"Hospital Expenditure: The Commissariat." 2s. 6d.

All these are published by THE SCIENTIFIC PRESS, Ltd., and may be obtained through any bookseller or direct from the publishers, 28 and 29 Southampton Street, London, W.C.

### Travel Notes.

By Sister Grace.

### GOOD FRIDAY IN SPAIN.

It was my good fortune to be close to the wonderful old Spanish town of Fuenterrabia two years ago in Holy week, and there to witness the very singular and picturesque services and procession with which they celebrate the great day of our Lord's Passion and death. These services are most impressive throughout Spain, but I have always understood that they are unusually striking in the little frontier town of Fontarabie as we are more accustomed to call it. Partly, this is owing, no doubt, to the singularly devout and simple character of the strange race of Basques who inhabit this part, and partly to the very marked and mediæval character of the town itself—so unlike any other that I have visited on the continent.

I was living in France at the time, but only just across the frontier, which is here marked by the Bidassoa, so often a bone of contention between the opposing forces in 1813. There are two ways of reaching Fontarabie from France: one a drive round by Irun, crossing a bridge which spans the Bidassoa, or, which is far preferable from an artistic point of view, taking boat from Hundaye.

This latter was our plan, having previously engaged the services of our special friend amongst the ferry-men. On this grand day he was resplendent in a spotless white shirt and trousers, the latter turned up to the knees, displaying his muscular brown legs, tanned with the scorching sun of this baking corner of the earth.

He was a tried and trusted friend and had ferried us over many times, and acted as interpreter when my meagre store of Spanish fell short of the demands made upon it, for, curiously enough, one stands almost with one foot in France here and yet no one understands French. The Spaniard, charming, courteous, and dignified as he is, is not a progressive man, the Spanish language being in his opinion (and he is not far wrong) the grandest in the world. Why unprofitably learn another?

It was a brilliant morning and the town was looking its best. The landing-stage is a green and slimy stone pier with crumbling sea-weed laden steps, so irregular and time-worn that extreme circumspection is advisable in venturing thereon; but, had we not our Elie, who would have suffered tortures rather than we should have encountered the slightest inconvenience.

One mounts gradually to a time-worn gate, black, grim, and narrow, and looking through one sees one of the most picturesque views in Europe; the little street, narrow and uneven, ascends to the church, which has something of a Moorish character with its almost unwindowed walls and round bell tower; then the street curves and is lost to view. It is easy to see how well such a groundwork would lend itself to the beauties of a religious procession.

The houses are immensely lofty and the numberless graceful balconies cling to the dark walls like beautiful parasites.

I could write long about Fontarabie, but must not linger, or I shall not have sufficient space to tell you of the service. We immediately entered the church to secure places and yet be sufficiently near the door to get out in time to squeeze into the narrow street to see the procession. The church was in almost absolute darkness, the few windows being obscured by black and scarlet hangings, the few blood-red rays permitted by the latter having a very weird effect.

There were various prayers and ceremonies, differing slightly from the Catholic ritual in France, and close to the high altar were three crosses with life-sized figures; these were obscured from view by something that appeared to me like a gauze curtain, to be removed later; after a long time and various ceremonies some thirty men tramped in, very correctly clothed, to represent the Roman soldiery; these grouped themselves around the crosses.

The organ gave forth mysterious and awe-inspiring music, especially when the remaining rays of light were obscured from the windows to represent "there was darkness over all the land!"

I cannot adequately express the very awe-inspiring effect of this total darkness, the subdued music, and the representation of the Crucifixion actually present. I confess I felt it so much that the sensation at one time was almost unbearable, and the desire to get out once more into the blessed light of day, all but too much for my self-control. However, it passed—a modicium of light was re-admitted, the veil was withdrawn, and we listened to an impassioned sermon delivered in sonorous Spanish. Then came the deposition and more prayers, and being close to the door, I slipped out without disturbing anyone and took up my position half-way down the main street. It was some time before the procession began, and the length was extraordinary—far greater than that of the entire street. We all reverently knelt as it passed us, and it certainly was deeply impressive.

I am not sure that I can remember the exact order in which it passed, but it was much as follows:—Various saints, such as St. Veronica, St. Francis, and St. Catherine, represented by life-size figures and usually clothed in real raiment, not simulated, were carried by on platforms something resembling huge trays. Some were borne by monks, especially Franciscans, in their brown habits, but many were supported by townsmen in evening dress and with white gloves; then these various pageants were divided by parties of thirty or forty children—very small girls dressed in white ball dresses and having wreaths on their heads; or by a similar number of men in ordinary dress and of all classes carrying mighty candles lighted and flaring. Then, I think, followed an enormous tray which required a vast number of supporters; they were Franciscans, and one could see the strain was very great. On this large platform was represented the agony in the garden with the sleeping disciples somewhat in the background. The next pageant was that of the Marys. The figure of the Blessed Virgin was extremely good, that of St. Mary Magdelene more theatrical. There was considerable difference in the artistic merit of the figures.

Next came a striking group of male penitents, all clothed alike in one simple shapeless garment, something like a scanty surplice which reached to just below the knee, leaving the legs and feet bare. It was of an indefinite purple colour, and confined at the waist by a cord. Each penitent carried a cross over his shoulder, and kept his eyes on the ground.

Immediately behind these came a figure of Our Lord carrying His cross, and this, which one would fain have had the best of all, fell sadly short of artistic pre-eminence. The figure itself was well conceived, but the devout originators committed the error of clothing "The Man of Sorrows" in a rich purple velvet robe!

The procession was concluded by a wax figure of Our Blessed Lord (the same which had been taken down from the cross), carried recumbent in a glass case.

The effect of this vast procession passing through the old streets and along the ramparts within living walls of kneeling humanity was more striking than I can express, and dwells in my memory as one of the events of my life. In many ways it was infinitely more remarkable than Holy week in Seville, because the simplicity and devotion of the crowd was entirely unmarred by hordes of gaping and, alas! often irreverent sightseers. Probably we were the only strangers in the crowd, and I feel morally certain that we were the only English present.

### TRAVEL NOTES AND QUERIES.

Ventimiglia (Beta).—Ventimiglia is a small town with the houses lying close together. There are three modest inns. I should recommend the Hôtel Suisse. Probable cost (by arrangement beforehand) about 6 fr. per day, which is 5s. If you know anything of hotel life and travelling, you may arrange for less. I do not quite know why you chose Ventimiglia for residence. If your tastes are artistic, well and good; it is most picturesque; but it is not a place for a delicate person or one who likes any gaiety. Let me hear further if I can help you in other ways.

